# The potential diagnosis role of TP53 mutation in advanced bladder cancer: A meta‐analysis

**DOI:** 10.1002/jcla.23765

**Published:** 2021-03-29

**Authors:** Yihao Liao, Huiqin Tang, Miaomiao Wang, Keke Wang, Youzhi Wang, Ning Jiang

**Affiliations:** ^1^ Tianjin Institute of Urology The Second Hospital of Tianjin Medical University Tianjin China

**Keywords:** bladder cancer, meta‐analysis, mutation, TP53

## Abstract

**Background:**

Bladder cancer is one of the most common urological cancers all over the world, and NMIBC occupies almost 80% of recently diagnosed bladder cancer cases. Progress and recurrence of bladder cancer are the main problems during the disease. The level of TP53 mutation is obviously higher in the high stage than the lower. This meta‐analysis is to evaluate the potential diagnosis feature of TP53 mutation by the expression of TP53 mutation of Ta stage vs high stage in bladder cancer.

**Methods:**

A systematic search of databases was conducted, and some relevant articles were selected. Next, the meta‐analysis was carried out according to the standard guidelines.

**Results:**

There were seven researches in which 677 participants were selected at the basis of inclusion standard. TP53 mutation was associated highly with increased diagnosis of bladder cancer. We found that the high stage of bladder cancer has obviously higher level of TP53 mutation than the lower stage, and these patients of MIBC have higher expression of TP53 mutation compared with NMIBC. No significant publication bias has been observed in this meta‐analysis. The expression of TP53 mutation might be a diagnose‐related biomarker for lots of patients with bladder cancer.

**Conclusions:**

The results of this meta‐analysis provided further evidences that the expression of TP53 mutation was associated with the diagnosis efficiency of advanced bladder cancer. Higher expression of TP53 mutation was observed in the high stage of bladder cancer or the MIBC, and lower expression of TP53 mutation in the Ta stage of bladder cancer or the NMIBC. The expression level of TP53 mutation was probably a critical diagnosed biomarker in advanced bladder cancer.

## Graphical abstract

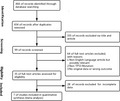

## 1 INTRODUCTION

Bladder cancer is one of the most common urological cancers all over the world; it ranks the fourth in male patients and eleventh in female patients in terms of its morbidity among all kinds of tumors.^1^ According to the statistical analysis in 2021,^2^ there were almost 83,730 new patients who were diagnosed with bladder cancer in America. It was interested that the percentage of male patients was three times more compared with the percentage of female patients, and in which smoking might be the main cancer‐promoting factor for those male patients with bladder cancer. The results of analysis further showed that there were approximately 17,200 deaths derived from bladder cancer, and the death rate of male patients was twice more than female patients.^3^ Furthermore, previous studies have identified that smoking, chemical materials, age, diet, chronic inflammation, and infection of bladder were the main risks factors for the occurrence of bladder cancer.^4^, ^5^ It has been known that bladder cancer was mainly divided into two types, including non‐muscle invasion bladder cancer (NMIBC) and muscle invasion bladder cancer (MIBC).^6^ NMIBC accounted for almost eighty percent in newly diagnosed bladder cancer patients according to the newly research statistics.^7^ Although the prognosis of patients with NMIBC has obtained great improvements, however, the rates of progression and recurrence were still the biggest problem; there were almost more than 60% of NMIBC patients would recur and more than 20% would deteriorate to a higher stage.^8^ Nieder AM et al^9^ had found that bladder cancer with stage T1 presented invasion of the lamina propria accounting for 25% of NMIBC. There were approximately a half of T1 stage NMIBC who were treated with intravesical therapy would develop to MIBC within five years.^10^ So, many patients were undergoing huge economic and psychological stress because of bladder cancer. However, we have no idea about when NMIBC would progress to MIBC, an effective approach is to seek a newly and sensitive biomarker to predict the probability of progression. Currently, bladder cancer was only detected by cytology and cystoscopy, while the two techniques both have their own limitations: cystoscopy was a surgical intervention that would cause patient's discomfort and cytology's low sensitivity. It was critical to diagnose bladder cancer at an early stage of disease (pTa‐pT1 according to TNM stage), it would be very serious and tricky; once it developed to more aggressive high stage (pT2‐4). It was known that some available markers for bladder cancer diagnosis mainly contained BTA Stat, BTA Trak, NMP22,^6^ Bladder Check, the ImmunoCyt test, UroVysion test, BLCA‐4, Survivin, and Microsatellite; however, their application to clinical practice still has many limitations due to their high false‐positive rate for high‐stage bladder cancer verification. Furthermore, these tests were not accurate enough to be used for detecting or screening the early stage of bladder tumors, especially in advanced bladder cancer.^11^ It has been reported that various cell cycle modulators, such as TP53, CCNB1, p27, and p16, were frequently deregulated in bladder cancer.^12^, ^13^ And tumor protein p53 (TP53) was recognized as a tumor suppressor factor for several common tumors, which could regulate the expression of lots of critical genes in order to further play multiple roles in cope with complicated cellular processes.^14^ The mutation of TP53 was frequently observed in lots of patients of bladder cancer,^15^, ^16^ and TP53 mutation might cause the progress of bladder cancer because TP53 and TP53 related pathways regulated many important carcinogenesis‐related signal pathways.^17^, ^18^ Previous researches had reported that TP53 mutation was positively associated with the progression of NMIBC.^19^, ^20^ To further identify the potential role of TP53 mutation in the progress from Ta stage to more high stages (or from NMIBC to MIBC), we conducted a meta‐analysis to explain the importance of TP53 mutation for diagnosis in advanced bladder cancer.

## 2 METHODS

This article was a meta‐analysis, in which abstract information from the published articles was to make further statistical analysis. Institutional Review Board approval was not needed for this study. Our meta‐analysis was performed in the light of the Preferred Reporting Items for Systematic reviews and Meta‐Analyses (PRISMA) guidelines.^21^

### 2.1 Search strategy

We engaged an exhaustive literature review with search terms (Table 1) with English only. We restricted the search keywords “TP53 mutation and bladder cancer” from PubMed, Web of Science, Cochrane Library, EMbase, Chinese National Knowledge Infrastructure (CNKI) and Wanfang Databases, and the qualified published articles were selected by two reviewers.

**TABLE 1:** Characteristics of trials included in meta‐analyses

Study	Year	Country	Sample	Number	Ta		High‐stage		NMIBC		MIBC	
					WT	M	WT	M	WT	M	WT	M
M. Traczyk‐Borszynska	2016	Poland	Tissues	185	94	12	61	18	NA	NA	NA	NA
Takashi Kawahara	2019	Japan	Tissues/blood	103	NA	NA	NA	NA	51	8	22	22
Nicolas Noel	2015	France	Tissues/urine	103	27	18	15	32	NA	NA	NA	NA
Mahoor S Nanda	2011	Sher‐i‐kashmir	Tissues	60	33	6	9	12	35	4	11	14
Thorsten H. Ecke	2008	Germany	Tissues	75	35	11	12	15	NA	NA	NA	NA
Thorsten H. Ecke	2008	Germany	Sediment cells/tissues	75	31	25	3	16	31	25	3	16
Nadina Erill	2004	Spain	Tissues	76	27	6	18	17	NA	NA	NA	NA

### 2.2 Inclusion criteria

Researches were suitable qualified for inclusion when they meet the following criteria: (a) patients were pathologically verified to have bladder cancer; (b) either Ta stage or higher stage bladder cancer; (c) either NMIBC or MIBC; and (d) studies involve TP53 mutation.

### 2.3 Exclusion criteria

Researches were precluded due to any of the following criteria: (a) Studies were published repeatedly; (b) complete data cannot be obtained from articles or authors; and (c) laboratory researches such as some studies involved with animal models or bladder cancer cell lines.

### 2.4 Data extraction

This meta‐analysis was performed in light with the Preferred Reporting Items for Systematic Reviews and Meta‐Analyses (PRISMA). Based on the above inclusion criteria, information was independently abstracted by reviewer for each qualified research. Study characteristics including the name of first author, country of included subjects, year of publication, the number of bladder cancer cases, the source of samples, cases number of TP53 mutation in Ta stage and high‐stage bladder cancer, and the cases number of TP53 mutation in NMIBC and MIBC.

### 2.5 Statistical analysis

The main outcome measures for this meta‐analysis were percentage of TP53 mutation in Ta stage and high‐stage bladder cancer and in NMIBC and MIBC. With the obvious results we have analyzed from this meta‐analysis, we could use the percentage of TP53 mutation to predict the possibility of progress in bladder cancer. For dichotomous data, a summary risk ratio (RR) and its 95% confidence interval (CI) were counted. Our analysis has no heterogeneity due to our analysis 95% CI = 0, we did not conduct subgroups analysis. A visual inspection of funnel plots was used to explain whether there is a publication bias. This meta‐analysis was conducted rely on Review Manager (RevMan) software version 5.3 (RevMan 5.3).

## 3 RESULTS

### 3.1 Records searching

**FIGURE 1:**
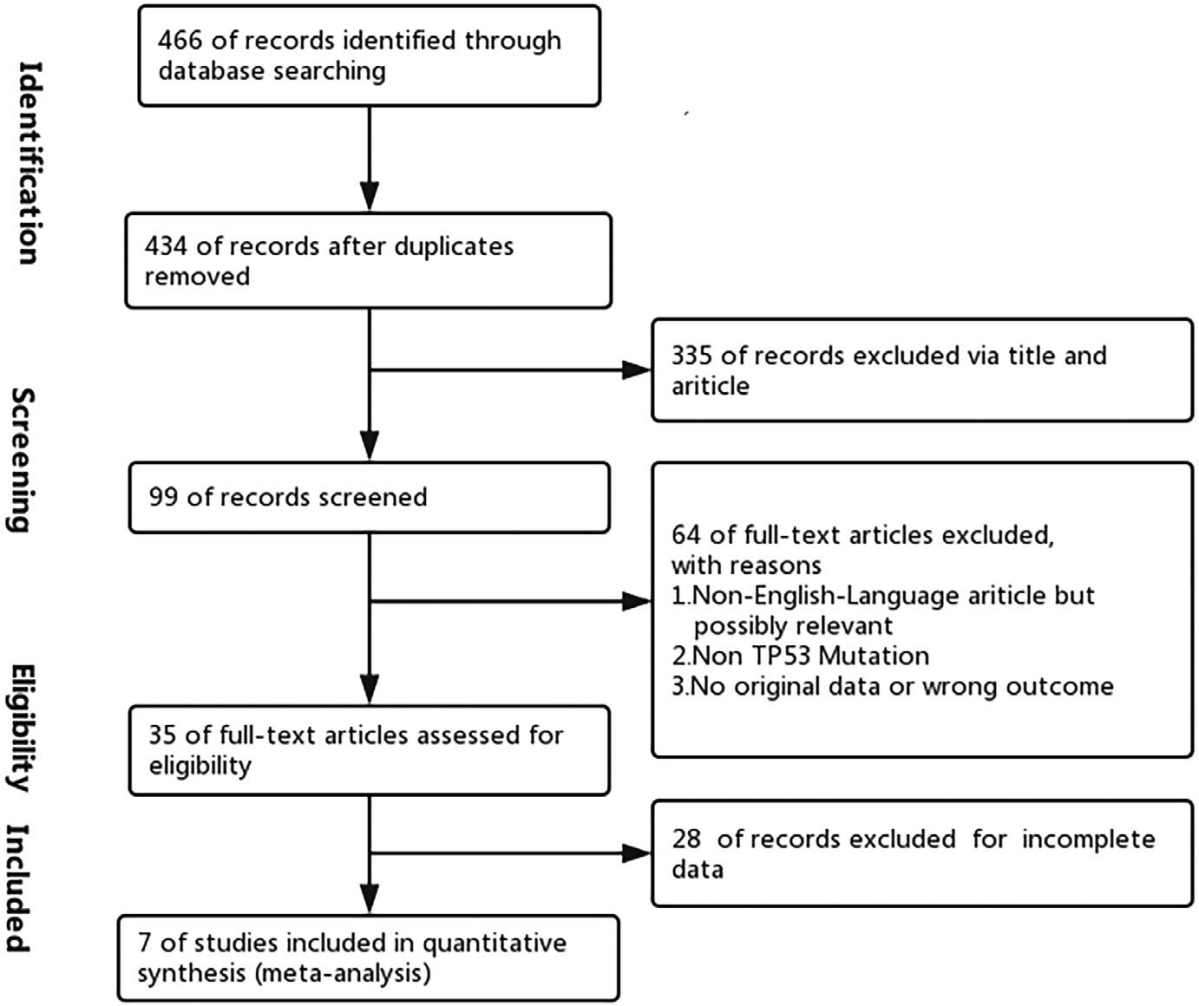
Flowchart of selecting process for meta‐analysis

Through careful screening, 99 relevant articles consistent with our research aim. Sixty‐four articles of which were excluded after reading full text, and 7 articles were finally included in addition to the remains which have no complete data about TP53 mutation in bladder cancer. The flowchart of selecting process and the excluded reasons of studies is integrated in Figure 1. Altogether, 7 researches were incorporated in this meta‐analysis.^22^, ^23^, ^24^, ^25^, ^26^, ^27^, ^28^

### 3.2 Included study characteristics

The included studies were published from 2004 to 2019. Studies revolve different stage bladder cancer, a part of studies research TP53 mutation in the Ta stage and high‐stage bladder cancer, and the other part research in NMIBC and MIBC. The details were listed in Table 1.

### 3.3 Sensitivity analysis and publication bias

Meta‐analysis of the correlation between TP53 mutation with progress in advanced bladder cancer resulted in *p* < 0.00001 and I^2^ = 0%, presenting that there was no heterogeneity, and the sensitivity analysis and the subgroup analysis did not be carried out. And there is no publication bias based on the funnel chart (Figure 3).

### 3.4 The relationship of TP53 mutation and some cancers

The high percentage of TP53 mutation in exons 5–8 encoding the DNA‐binding domain has been clearly known quite a long time.^29^ Although some deviations occur during our analysis, the truth of the high ratio of TP53 mutation in various cancers is definite.^30^ The distribution of TP53 mutation is distinctive in all cancer‐relative genes, including tumorigene and tumor suppressor gene. The TP53 mutation is being observed frequently in many cancers, such as non‐small cell lung cancer,^31^ chronic lymphocytic leukemia,^32^ acute myeloid leukemia, ^33^ and breast cancer. Thorsten Zenz had found that chronic lymphocytic leukemia patients with TP53 mutation usually have a poor prognosis, and TP53 mutation would affect the treatment effect of CLL patients. Other researches observed that different molecular subtype breast cancer with TP53 mutation would have different clinical prognosis and identifying the new role of TP53 in the progression of breast cancer.^34^ Many researches also found that TP53 mutation was usually found in various cancers or other non‐cancer diseases, which remind us to further elaborate the importance of TTP53 mutation in various life activities and processes.

### 3.5 Meta‐analysis results

Based on the findings from above researches, we have found that the expression of TP53 mutation was closely related with the progression of many cancers. To explore the role of TP53 mutation in bladder cancer and the relationship between TP53 mutation and the progression of bladder cancer and then further understand the role of TP53 mutation in muscle invasion bladder cancer and non‐muscle invasion bladder cancer, we conduct a briefly meta‐analysis to explain the specific relationship.

**FIGURE 2:**
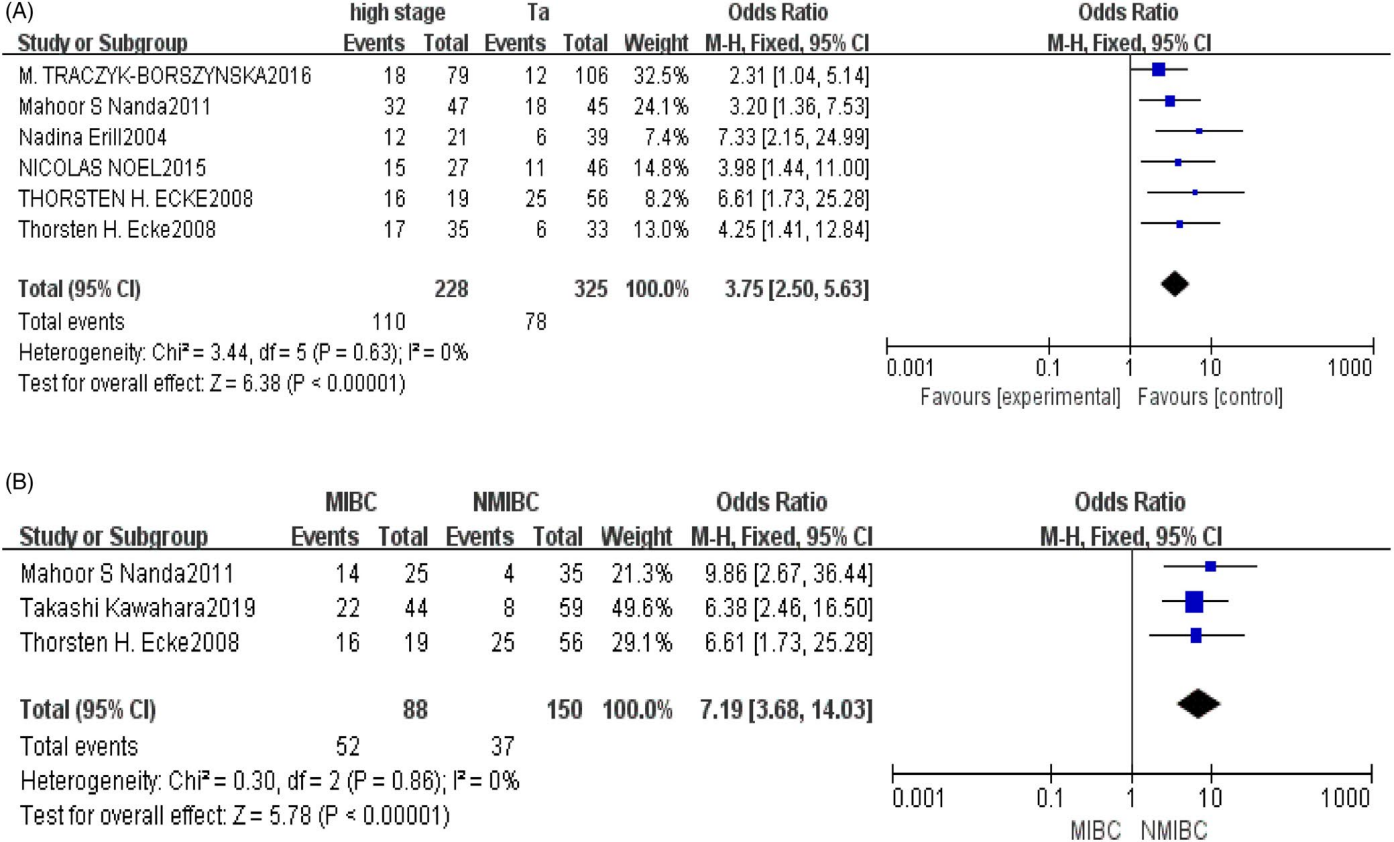
Forest plot for the change with TP53 mutation. (A) Forest plot for the comparison the expression of TP53 mutation in high‐stage bladder cancer to Ta stage bladder cancer. CI, confidence interval; M‐H, Mantel‐Haenszel method. (B) Forest plot for the comparison the expression of TP53 mutation in MIBC to NMIBC. CI, confidence interval; M‐H, Mantel‐Haenszel method; MIBC, muscle invasion bladder cancer; NMIBC, non‐muscle invasion bladder cancer

**FIGURE 3:**
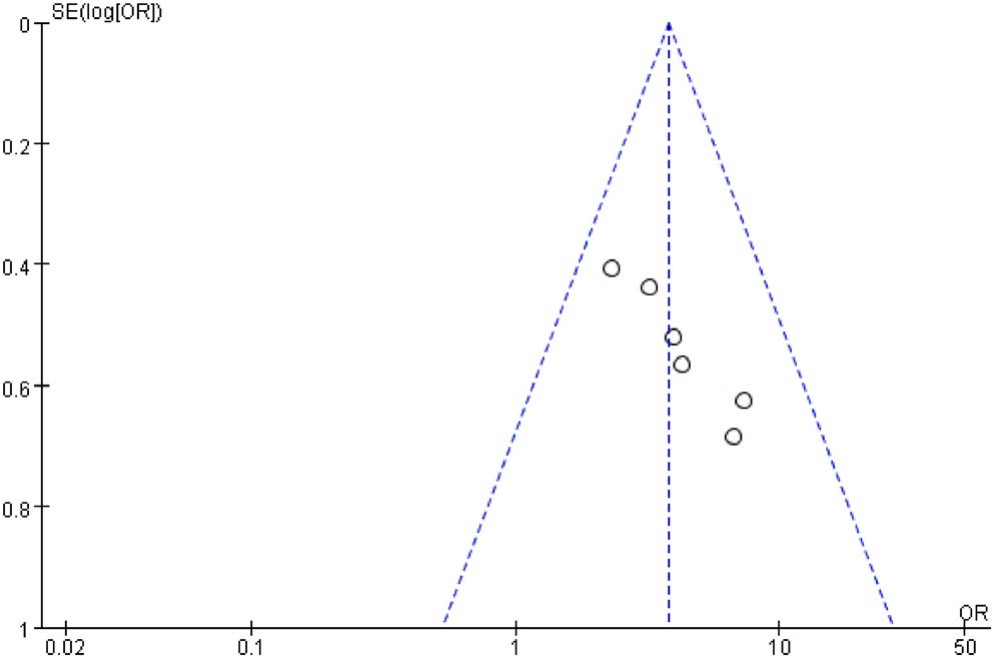
Publication bias determination using funnel plot. OR, odds ratio; SE, standard error

The pooled result revealed that the expression of TP53 mutation was positive associated with the high‐stage bladder cancer compared with Ta stage (OR = 3.75; 95% CI, 2.50–5.63; *p* < 0.00001; Figure 2A). Funnel plot did not show any skew, (I^2^ = 0%; *p* < 0.00001) which suggests no any publication bias exists (Figure 3).

Considering the expression of TP53 mutation has an effect on NMIBC and MIBC, we try to explore the potential role of the level of TP53 mutation in MIBC compared with NMIBC, and we found that TP53 mutation was markedly higher in MIBC than in NMIBC (OR = 7.19; 95% CI, 3.68–14.63; *p* < 0.00001; Figure 2B). There was also no significant publication bias observed.

All above results reminded us that we can more likely to discover progress with Ta stage bladder cancer or NMIBC when expression of TP53 mutation markedly elevates. The expression of TP53 mutation is very likely to be a new biomarker for diagnosis of bladder cancer and the treatment target of bladder cancer. It may bring many improvements in the diagnosis and treatment history of bladder cancer.

## 4 DISCUSSION

As of now, cancer is still the second lethal reason preceded only by cardiovascular diseases in America and almost 599,108 people died due to cancer every year in the United States.^35^ According to American cancer statistic in 2021, there were approximately 83,730 new bladder cancer cases among which male bladder cancer patients accounted for 76% and female bladder cancer patients accounted for 24%, and almost 17,200 patients died due to bladder cancer.^36^ The morbidity of bladder cancer in American men ranked fourth and the mortality rate of bladder cancer ranked eighth among all kinds of cancers. Previous studies implied that bladder cancer mainly includes two species, containing non‐muscle invasion bladder cancer (NMIBC) and muscle invasion bladder cancer (MIBC), and NMIBC was known to have a good prognosis.^37^ It is essential to find out the suitable opportunity of NMIBC developing into MIBC. Other researches also showed that NMIBC accounted for almost 75%–85% (of which, 70% are Ta, 20% T1, and 10% carcinoma in situ lesions); however, 10%–30% T1 or carcinoma in situ patients progressed to MIBC within 5 years.^38^, ^39^, ^40^ Previous studies have proved that the 5‐year survival rate of patients was 94%. When patients were firstly diagnosed as bladder cancer at a lower stage, we should take positive measures to deal with the early stage of bladder cancer such as transurethral partial resection of bladder tumor or early bladder infusion chemotherapy.^41^ Most patients have a good prognosis and a long overall survival after treatment, but many bladder cancer patients lost many chances of receiving treatment when bladder cancer began to progress into muscle invasion. We have no idea about when NMIBC would develop into MIBC, so an effective marker was urgent to further predict the progression with lower stage of bladder cancer.^37^ TP53 was known as a critical role in the cell apoptosis and the regulation of cell cycle, and many researchers have found that the main function of TP53 depended on its phosphatase activity. Major antagonism, the PI3K/AKT signal pathway, and lots of non‐AKT relative functions are also being gradually valued. While TP53 mutation has been observed in various kinds of cancers recently, it was found to be tightly associated with the occurrence and development of various cancers. Previous researchers had found that the development of bladder cancer was clearly related with the mutations of FGFR3 and TP53 genes.^42^, ^43^ Fei Mao et al^41^ identified that Leptin G19A polymorphism was related with a decreased risk of bladder cancer in Chinese Han people, which implied that the mutation of various genes was associated with the occurrence and development of bladder cancer. M. Traczyk‐Borszynska^22^ and his teammates confirmed that TP53 mutations were more common in clinically and histologically advanced disease compared with FGFR3 mutation. In addition, their results also confirmed that TP53 mutations were an unfavorable prognostic factor for several cancers, and any changes in this gene were closely associated with poor differentiation, poor prognosis and advanced UCC.^26^, ^43^ According to above researches, we have found that TP53 mutation and some other important gene mutations could influence multiple cancer‐relative signal pathway of bladder cancer and finally promoted the progress and development of bladder cancer, which made TP53 mutation a potential and newly therapeutic target. However, there is no any clear molecular evidence directly confirm or deny that the progression from NMIBC to MIBC has been changed.^44^ Schlechte HH et al. found that the percentage of TP53 mutations was observed in NMIBC in the range of 35%, while the percentage of TP53 mutations increased frequencies up to 70% in MIBC.^45^ However, there is no article explaining the unequivocal association between TP53 mutation and progression of Ta stage bladder cancer or NMIBC.

In this meta‐analysis, we tried to explore the association between the expression of TP53 mutation and the progression of Ta stage bladder cancer or NMIBC in seven studies. After conducting meta‐analysis, the results showed that the expression level of TP53 mutation was significantly associated with increased diagnosis efficiency of the occurrence of Ta stage bladder cancer(OR = 3.75; 95%CI, 2.50–5.63; *p* < 0.00001). The expression of TP53 mutation had a strong relevance with increased diagnosis efficiency with MIBC (OR = 7.19; 95%CI, 3.68–14.63; *p* < 0.00001). The overall analysis provided strong supports to the initial findings, which further confirmed the potential diagnosis role of TP53 mutation in advanced bladder cancer. Detecting the levels of TP53 mutation might be applied to clinical practice to observe the early progression of Ta stage bladder cancer or NMIBC. A good prognosis and a longer progress‐free survival were most likely obtained when the detecting of TP53 mutation expression was used to diagnose the progress with bladder cancer.

However, some un‐provided parameters are probably associated with progression of bladder cancer because of our based unadjusted estimated results. Some Intrinsic properties might affect the presented results. Several limitations should be noticed in this meta‐analysis. First of all, to further illustrate our conclusion, a more and larger bladder cancer samples with TP53 mutation need to be included in our research. Secondly, the samples involved in our research are from different bladder cancer patients who were treated with different drugs and manners, which made the results of this study exists partly bias. Finally, only these articles of English language were Incorporated in this meta‐analysis, thus further researches of multiple languages should be included to further confirm our results. Therefore, the results should be interpreted cautiously.

## 5 CONCLUSIONS

Our comprehensive meta‐analysis brought to light that the expression of TP53 mutation was obviously related to progression with Ta stage or NMIBC, TP53 mutation was probably a potential diagnosis marker in advanced bladder cancer. However, to further identify our conclusion about the diagnosis role of TP53 mutation in advanced bladder cancer, more and larger prospective studies are needed to be conducted.

## CONFLICT OF INTEREST

The authors have no conflicts of interest to declare.

## AUTHOR CONTRIBUTIONS

Yihao Liao involved in project development, data collection, data analysis, and manuscript writing. Huiqin Tang involved in data collection. Miaomiao Wang involved in data analysis. Keke Wang involved in manuscript writing. Youzhi Wang involved in data collection. Ning Jiang involved in project development.

## RESEARCH INVOLVING HUMAN PARTICIPANTS AND/OR ANIMALS

This study does not involved in studies of human participants and/or animals.

## INFORMED CONSENT

Informed consent of all authors.

## Data Availability

All data and material are available.
